# Simultaneous blocking of the pan‐RAF and S100B pathways as a synergistic therapeutic strategy against malignant melanoma

**DOI:** 10.1111/jcmm.15994

**Published:** 2020-12-30

**Authors:** Ke‐Jia Wu, Shih‐Hsin Ho, Chun Wu, Hui‐Min D. Wang, Dik‐Lung Ma, Chung‐Hang Leung

**Affiliations:** ^1^ State Key Laboratory of Quality Research in Chinese Medicine Institute of Chinese Medical Sciences University of Macau Macao SAR China; ^2^ State Key Laboratory of Urban Water Resource and Environment School of Environment Harbin Institute of Technology Harbin China; ^3^ Department of Chemistry Hong Kong Baptist University Kowloon Tong Hong Kong; ^4^ Graduate Institute of Biomedical Engineering National Chung Hsing University Taichung Taiwan; ^5^ Graduate Institute of Medicine College of Medicine Kaohsiung Medical University Kaohsiung Taiwan; ^6^ Department of Medical Laboratory Science and Biotechnology China Medical University Taichung City Taiwan

**Keywords:** drug discovery, malignant melanoma, pan‐RAF, S100B‐p53 interaction, synergistic

## Abstract

Melanoma is a very aggressive form of skin cancer. Although BRAF inhibitors have been utilized for melanoma therapy, advanced melanoma patients still face a low five‐year survival rate. Recent studies have shown that CRAF can compensate for BRAF depletion via regulating DNA synthesis to remain melanoma proliferation. Hence, targeting CRAF either alone or in combination with other protein pathways is a potential avenue for melanoma therapy. Based on our previously reported CRAF‐selective inhibitor for renal cancer therapy, we have herein discovered an analogue (complex **1**) from the reported CRAF library suppresses melanoma cell proliferation and melanoma tumour growth in murine models of melanoma via blocking the S100B and RAF pathways. Intriguingly, we discovered that inhibiting BRAF together with S100B exerts a novel synergistic effect to significantly restore p53 transcription activity and inhibit melanoma cell proliferation, whereas blocking BRAF together with CRAF only had an additive effect. We envision that blocking the pan‐RAF and S100B/p53 pathways might be a novel synergistic strategy for melanoma therapy and that complex **1** is a potential inhibitor against melanoma via blocking the pan‐RAF and S100B pathways.

## 1 INTRODUCTION

Melanoma is a highly aggressive form of skin cancer with high resistance to chemotherapeutic drugs.^1^ While BRAF inhibitors have shown some improvement in survival for melanoma therapy, advanced melanoma patients are still faced with low five‐year survival rates.^1^, ^2^, ^3^, ^4^, ^5^, ^6^, ^7^ Marais *et al* reported that BRAF depletion can suppress MEK/ERK signalling to mediate p53‐dependent apoptosis in melanoma cells, whereas CRAF might compensate for BRAF depletion to maintain melanoma cell viability via regulating DNA synthesis.^8^ An in vivo study revealed that CRAF is needed for maintaining tumours in a mouse model of skin cancer.^9^, ^10^ Gray‐Schopfer *et al* reported that CRAF is required for melanoma cell proliferation and suggested that pan‐RAF inhibitors might be better than BRAF‐specific inhibitors for melanoma therapy.^1^ Although these studies do not completely validate CRAF, they suggest that CRAF could play an important role for melanoma therapy.^11^ Sorafenib is a multi‐kinase inhibitor that selective targets CRAF over other proteins or kinases and has been reported to induce apoptosis in melanoma cells.^10^ AZ628 is a new pan‐RAF inhibitor that targets CRAF with nanomolar potency and has been reported to specifically inhibit most tumour cells including melanoma cells.^12^ Overall, the case for targeting CRAF together with BRAF or other protein pathways is becoming increasingly compelling.^11^, ^13^ In this respect, Ir(III) complexes possess distinct geometries, and their properties can tailored to recognize specific protein binding surfaces via modification of their auxiliary ligands.^14^, ^15^, ^16^, ^17^, ^18^, ^19^, ^20^, ^21^ In a previous work, we have reported a CRAF‐selective iridium‐based inhibitor for renal cancer therapy.^22^ In this study, we report that an analogue (complex **1**) from the reported CRAF library suppresses melanoma cell proliferation and melanoma tumour growth in murine models of melanoma via blocking the S100B and RAF pathways. In addition, we demonstrate that blocking the BRAF and S100B pathways is a novel synergistic strategy to control melanoma growth via restoring p53 function.

## 2 MATERIALS AND METHODS

### 2.1 Flow cytometry protein interaction assay

After conjugating S100B protein with biotin to polystyrene beads in Ca^2+^ buffer,^23^ the mixture was incubated with FITC‐labelled human p53 peptide. Then, the FITC fluorescence of the complex was detected by flow cytometry.

### 2.2 Animal experiments

The animal experiments were approved by the Laboratory Animal Care and Use Committee (IACUC) of the Experimental Animal Center of the Biotechnology Center. All the animal experiments were performed at the Academia Sinica Common Animal Facility IACUC. NOD.CB17‐Prkdcscid/ NcrCrl (NOD/ SCID) female mice (6‐8 weeks) were purchased from Experimental Animal Center (BioLASCO, Taiwan Co., Ltd) and then were housed at temperature‐controlled room (22 ± 2°C, humidity 55% ± 10%), on a light/dark schedule with free access to food and water.

### 2.3 Melanoma xenograft assay

NOD/ SCID female mice at 8 weeks of age were injected with A375 cells. The injection site was sanitized using 70% ethyl alcohol. 1 × 10^6^ A375 cells were suspended in 0.1 mL of PBS and subcutaneously implanted into mice. The mice were observed for 7 to 10 days, until the tumour grew to an appropriate size (about 100 mm^3^). Then, complex **1** was administrated via subcutaneous injection to mice and the therapeutic effect on melanoma tumours was observed.^22^

### 2.4 Statistical analysis

For statistical analysis, data were analysed using one‐way analysis of variance (ANOVA) with GraphPad Prism 5.0.

## 3 RESULTS AND DISCUSSION

### 3.1 Complex 1 inhibits melanoma cell proliferation

The combination of targeting CRAF with other protein pathways is becoming increasingly compelling for melanoma therapy.^11^, ^13^ In a previous work, we have reported a potent selective CRAF inhibitor (CI) (Figure S1) for renal cancer therapy.^22^ To discover a potent inhibitor for melanoma therapy via targeting multiple key pathways including CRAF, we initially tested three analogues (iridium(III) complexes **1‐3**) and CI (Figure S1) that had exhibited measurable activity against CRAF in our previous work for anti‐melanoma activity.^22^ Intriguingly, complex **1** displayed the greatest cytotoxicity against two melanoma cell lines (A375 cells and A2058 cells), while CI showed only slight cytotoxicity under the same conditions (Table 1, Figure S2A,B). This result indicated that unlike with renal cancer, blocking the CRAF pathway alone insufficient to inhibit melanoma cell proliferation. We thus speculated that besides targeting CRAF, complex **1** might target another, possibly redundant, pathway to exert higher cytotoxicity in melanoma cells.

**Table 1:** In vitro cytotoxicity given as IC_50_ ± SD in μM of complexes **1**‐**7** and CI

Complex	A375	A2058	LO2
Complex **1**	0.16 ± 0.02	0.16 ± 0.05	>1
Complex **2**	0.26 ± 0.02	0.32 ± 0.03	>1
Complex **3**	>1	>1	>1
Complex **4**	>1	>1	>1
Complex **5**	0.35 ± 0.04	0.44 ± 0.01	0.21 ± 0.04
Complex **6**	0.21 ± 0.02	0.11 ± 0.05	0.12 ± 0.01
Complex **7**	>1	>1	>1
CI	>1	>1	>1

The previous study had indicated that both of C^N and N^N ligands were important for CRAF inhibition.^22^ In this study, replacing the N^N ligand of complex **1** led to a significant decrease in cytotoxicity of complexes **2** and CI, despite two of these complexes having the same C^N ligand as complex **1** (Table 1, Figure S2A,B). To enhance cytotoxicity against melanoma cells as well as retaining CRAF inhibition, we tested a set of reported iridium(III) complexes **4‐6** with the same C^N ligand as complex **1** while bearing with different N^N ligands (Figure S1). However, complexes **4‐6** were cytotoxic to the normal liver cell line LO2 with similar IC_50_ values to those against melanoma cells. In contrast, complex **1** displayed low cytotoxicity against LO2 cells (Table 1, Figure S2A, B, C). Hence, complex **1** was chosen as a candidate for further study against melanoma cell proliferation.

Rhodium(III) complexes have emerged as antitumour candidates.^14^, ^15^, ^16^, ^17^, ^18^, ^19^, ^24^, ^25^ To investigate whether the iridium(III) metal centre of complex **1** is important for inhibiting melanoma cell proliferation, the corresponding rhodium(III) congener of **1** was tested (Figure S1). However, the rhodium(III) complex **7** showed lower cytotoxicity than its iridium(III) counterpart **1**, despite sharing the same C^N and N^N ligands (Table 1, Figure S2A, B). This result demonstrates the critical role of iridium(III) metal centre in anti‐melanoma activity. In addition, the chemical stability results revealed that complex **1** was stable in acetonitrile/Tris‐HCl buffer (pH = 7.4) (8:2, v/v) solution and DMSO‐d6/D_2_O (9:1, v/v) solution for at least seven days (Figure S3). These results further encouraged us to investigate the applications and mechanisms of action of complex **1** against malignant melanoma.

### 3.2 Complex 1 blocks the MEK/ERK pathway and suppresses DNA replication via targeting BRAF and CRAF

**Figure 1:**
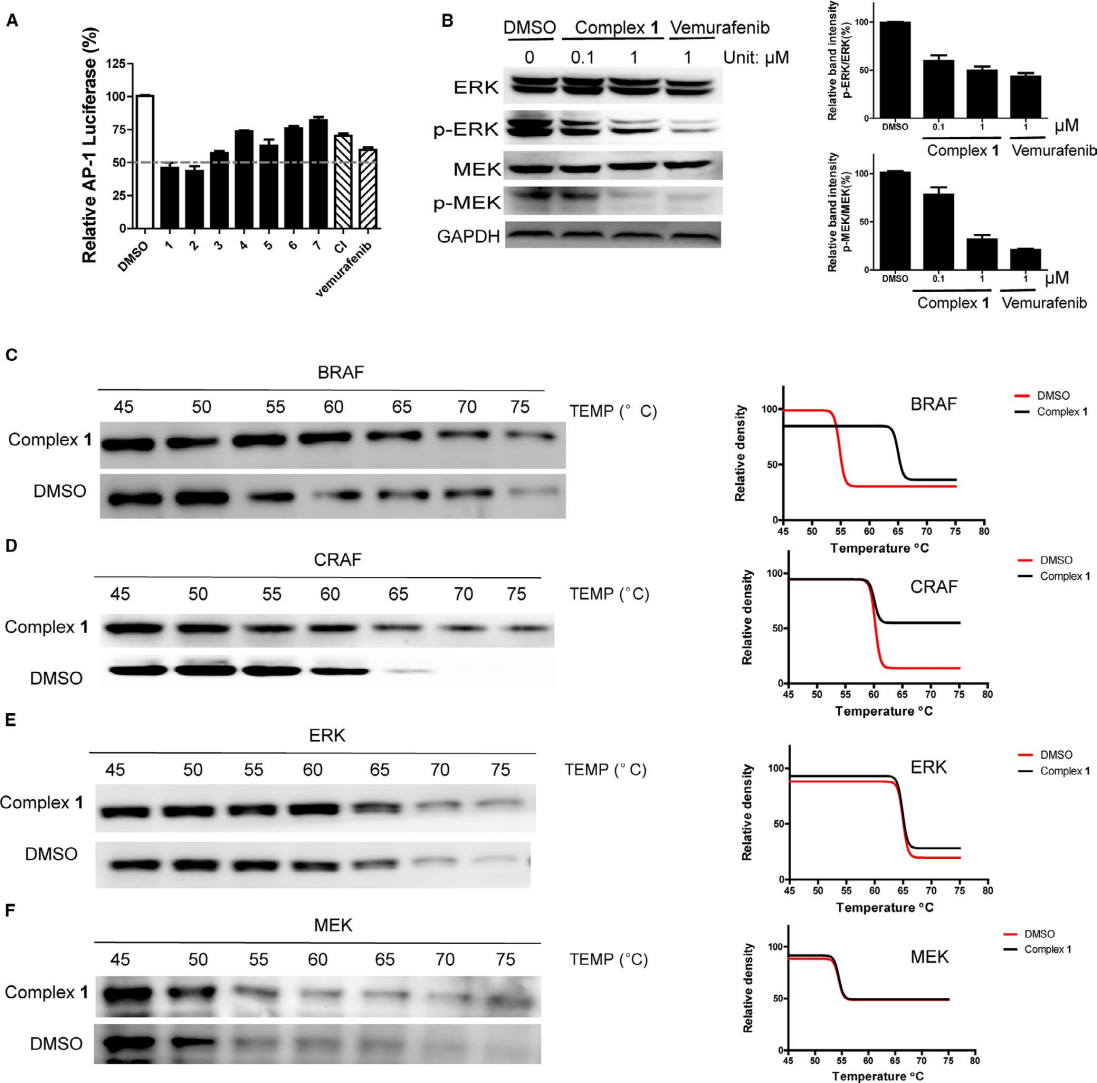
Complex **1** inhibits MEK/ERK pathway. (A) The effects of complex **1**‐**7** and CI on AP‐1 transcription activity. (B) The effects of complex **1** on MEK/ERK pathway. (C‐F) The effects of complex **1** on the thermal stability of BRAF, CRAF, MEK and ERK. Error bars represent standard deviation of the means of the results from three independent experiments

Accumulating evidence suggests that BRAF is necessary to maintain melanoma proliferation,^8^ while inhibiting the BRAF/MEK/ERK (MAPK) pathway has been reported as a potential approach for suppressing melanoma.^26^ Meanwhile, concomitant targeting of CRAF and BRAF has been suggested to be desirable for optimal antitumour activity in melanomas.^27^ To investigate whether the strong cytotoxicity of complex **1** against melanoma cells could be attributed to the dual inhibition of the BRAF/MEK/ERK pathway along with CRAF, we compared the effects of complexes **1‐7** and CI on the transcriptional activity of AP‐1, a primary downstream transcription factor of the BRAF/MEK/ERK pathway.^1^, ^28^, ^29^, ^30^ Intriguingly, complex **1** displayed a stronger inhibition of AP‐1 transcription activity than CI in melanoma cells (Figure 1A). This indicated that besides inhibiting CRAF activity, the BRAF/MEK/ERK pathway might act as a redundant pathway of complex **1** to regulate cell viability. To further investigate the action of **1** on the MEK/ERK signalling pathway in cells, an immunoblotting assay was performed. Complex **1** strongly inhibited MEK1/2 and ERK1/2 phosphorylation, but had no effects on the expression of total MEK1/2 and ERK1/2 (Figure 1B). The cellular thermal shift assay (CETSA) is used to study the thermal stabilization of proteins by ligands in cells or cell extracts.^31^, ^32^ The principle of CETSA is based on the fact that when ligands bind to a protein, the thermal stability of the protein‐ligand complex increases compared to the free protein alone, which is reflected by an increase in the melting temperature. To investigate the potential targets of complex **1** in the MEK/ERK signalling pathway, CETSA was performed. The results showed that complex **1** significantly stabilized BRAF and CRAF over MEK and ERK protein in A375 cell lysates compared to DMSO‐treated control (Figure 1C‐F). Performing an isothermal dose‐response fingerprint (ITDRF_CETSA_)^31^ revealed that complex **1** bound to BRAF and CRAF with *K*_d_ values of 0.9 ± 0.1 µM and 1.1 ± 0.1 µM respectively (Figure S4A). Taken together, these results indicate that complex **1** is a potent inhibitor of the MEK/ERK pathway *in cellulo*, presumably via binding to BRAF and CRAF proteins.

BRAF depletion can suppress the MEK/ERK pathway (while CRAF depletion has less effect on ERK activity in melanoma cells).^8^ To further verify that complex **1** targets the MEK/ERK pathway via engaging BRAF, siRNA was used to knockdown BRAF in A375 cells. As shown in Figure S4B, complex **1** treatment phenocopied BRAF knockdown in terms of inhibition of MEK1/2 and ERK1/2 phosphorylation in A375 cells. This indicated that the inhibition of MEK/ERK pathway by complex **1** might be partly attributed to the inhibition of BRAF activity. To further investigate the inhibition activity of complex **1** on BRAF, we evaluated the stability of p53 protein which is regulated by BRAF.^33^ The results showed that complex **1** can suppress p53 degradation in melanoma cells, which is phenocopied by BRAF knockdown, suggesting that the suppression of p53 degradation might also be partially attributed to the inhibition of BRAF activity by complex **1** (Figure S4C). Overall, these experiments indicate that BRAF is a potential target of complex **1** to exert its function against the p53 and MEK/ERK pathways. CRAF depletion reduces DNA synthesis and induces cell death in melanoma cells.^8^ To investigate whether complex **1** can suppress DNA replication via engaging CRAF, we evaluated the effects of complex **1** on DNA synthesis via the 5‐ethynyl‐2’‐deoxyuridine (EdU)‐labelling method.^34^ The results revealed that complex **1** treatment phenocopied the CRAF knockdown group (Figure S4D) in terms of blocking DNA synthesis. Taken together, these results demonstrate that complex **1** can inhibit the MEK/ERK pathway and suppress p53 degradation via inhibiting BRAF activity as well as suppressing DNA replication via suppressing CRAF activity. Our results also suggest that blocking the pan‐RAF pathway might be an effective strategy for melanoma therapy.

### 3.3 Complex 1 inhibits the S100B/p53 pathway

**Figure 2:**
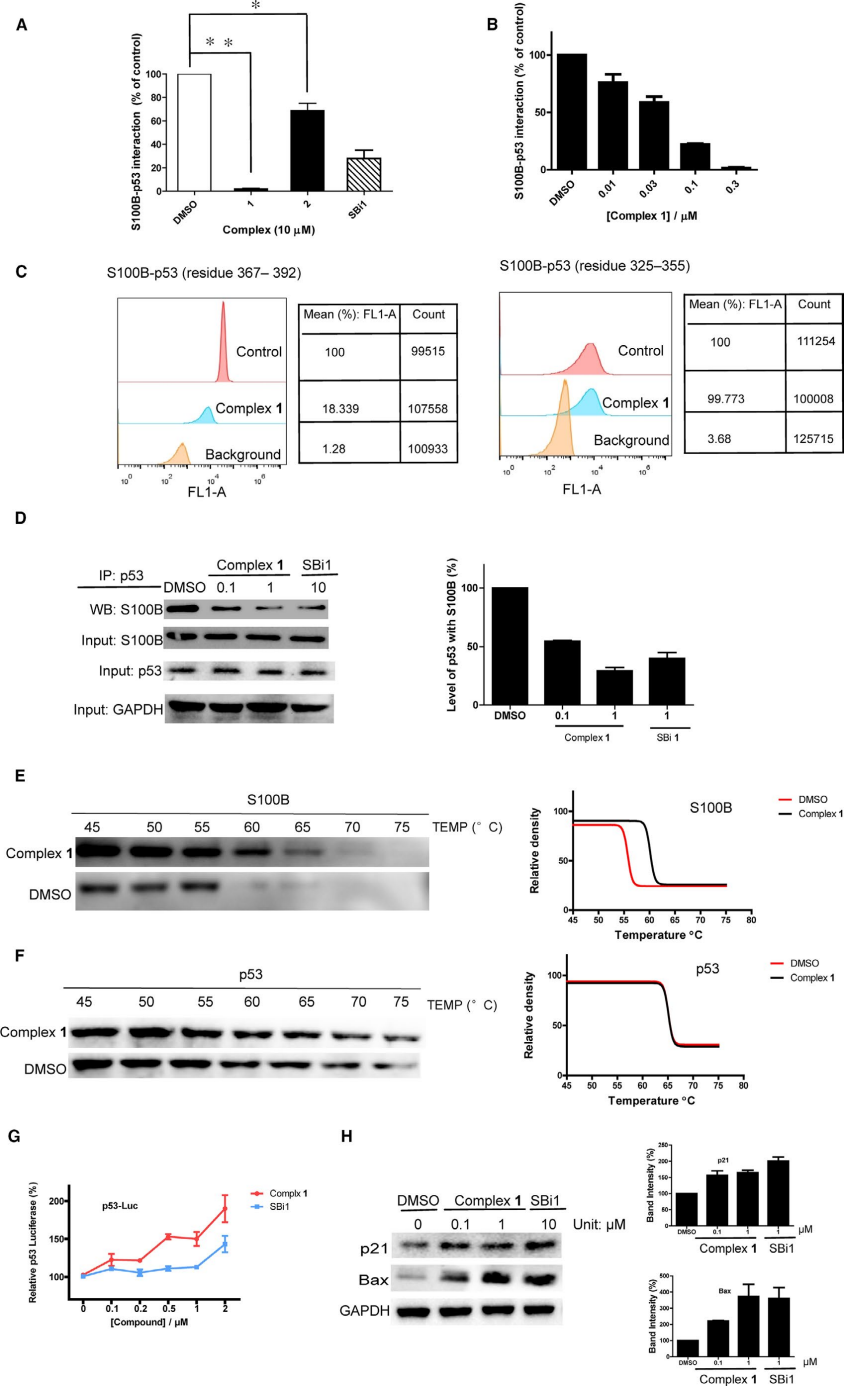
Complex **1** disrupts the S100B‐p53 interaction to restore p53 function by blocking S100B protein. (A) The effects of complexes **1‐2** and SBi1 (10 μM) on the S100B‐p53 interaction. (B) EC_50_ of complex **1** on the S100B/p53 PPI by FCPIA. (C) Effects of complex **1** (10 μM) at disrupting the interaction between S100B with p53^367‐392^ and p53^325‐355^ peptides by FCPIA. (D) Effects of complex **1** at inhibiting the S100B/p53 PPI in A375 cells as determined by Co‐IP. The protein samples were detected by Western blotting, and the band density was analysed. (E) Effects of complex **1** on the thermal stability of S100B. (F) Effects of complex **1** on the thermal stability of p53. (G) Effects of complex **1** or SBi1 on p53 transcription activity as detected by the luciferase assay. (H) Effects of complex **1** on p53 downstream protein expression by Western blotting. The protein samples were detected by Western blotting, and the band density was analysed. Error bars represent standard deviation of the means of the results from three independent experiments

S100B is overexpressed in malignant melanoma patients, and its expression directly correlates with the degree of malignancy.^5^ Impeding the interaction between S100B and the C‐terminal domain of p53 to restore the anti‐apoptotic function of p53 is an emerging approach for malignant melanoma therapy.^35^, ^36^, ^37^, ^38^, ^39^, ^40^ In a previous work, we have discovered that complex **2** exhibits S100B/p53 activity.^41^ Considering that complex **1** is structurally similar to complex **2**, differing only in the nature of its N^N ligands, we wished to investigate whether the higher cytotoxicity of complex **1** could also be partly attributed to its S100B/p53 inhibitory activity. The effects of complexes **1**‐**2** on S100B/p53 PPI inhibition was monitored by a flow cytometry protein‐protein interaction assay (FCPIA). The results revealed that complex **1** shown a greater inhibition of the S100B/p53 interaction than complex **2** (Figure 2A), with an EC_50_ value of 0.053 ± 0.004 μM (Figure 2B). This result revealed that complex **1** might act through inhibiting the S100B/p53 protein‐protein interaction in order to exert higher cytotoxic activity than complex **2**. Literature studies have suggested that besides binding to the C‐terminal domain of p53, S100B can also bind to the tetramerization domain of p53 to block p53 function.^36^, ^42^ To further investigate the action of complex **1** on the S100B/p53 interaction, we tested the ability of complex **1** to disrupt the interaction between S100B with the tetramerization domain (residues 325‐355) or the C‐terminus (residues 367‐392) of p53. Complex **1** inhibited the interaction between S100B with the C‐terminus and tetramerization domain of p53 by about 98% and 25% respectively at 10 μM (Figure 2C), indicating that complex **1** preferentially disrupts the interaction between p53 C‐terminal domain with S100B protein. The ability of complex **1** to disrupt the S100B/p53 interaction *in cellulo* was confirmed using a co‐immunoprecipitation (Co‐IP) assay (Figure 2D). Meanwhile, a cellular thermal shift assay^32^ in A375 cell lysates results revealed that complex **1** preferentially engaged S100B protein (*K*_d_ = 18.6 μM) over p53 protein (Figure 2E,F, S4A). ICP‐MS results revealed that complex **1** localized in the cytoplasm (Figure S5A). Overall, these results indicate that complex **1** can impede the interaction between S100B with C‐terminus of p53 via targeting the S100B protein in the cytoplasm.

Disrupting the S100B and p53 protein‐protein interaction leads to the induction of p53 transcriptional activity at targets including Bax and p21.^36^ To further investigate the function of complex **1** on the S100B/p53 pathway, the luciferase reporter assay and an immunoblotting assay were performed. The results showed that complex **1** increased p53 transcriptional activity (Figure 2G) and phenocopied S100B knockdown (Figure S5B) in terms of up‐regulating p21 and Bax protein levels in A375 cells (Figure 2H). The results suggested that complex **1** can restore p53 function, presumably via its ability to target S100B protein and disrupt its interaction with the C‐terminal domain of p53 in the cytoplasm.

As p53 plays a major role in regulating malignant cells, restoring p53 function would induce malignant cells apoptosis.^43^ To investigate the effects of complex **1** on melanoma cell apoptosis, the levels of pro and anti‐apoptotic proteins (Puma, PARP and Mcl‐1) in treated cells were evaluated. The results showed that complex **1** can induce PARP and Puma protein expression, while down‐regulating Mcl‐1 protein expression (Figure S5C). The apoptosis results were further confirmed by a flow cytometry assay (Figure S5D). The population of late‐apoptotic annexin V/PI double‐stained cells (Annexin V+/PI+) increased after treatment with complex **1** at 1 μM for 12 h, indicating that complex **1** can induce apoptosis in treated cells. Taken together, these results indicated that complex **1** can induce apoptosis in melanoma cells, which might be associated with the restoration of p53 function leading to modulation of pro‐apoptotic and anti‐apoptotic proteins.

### 3.4 Complex 1 acts via synergistically targeting S100B and BRAF in melanoma cells

**Figure 3:**
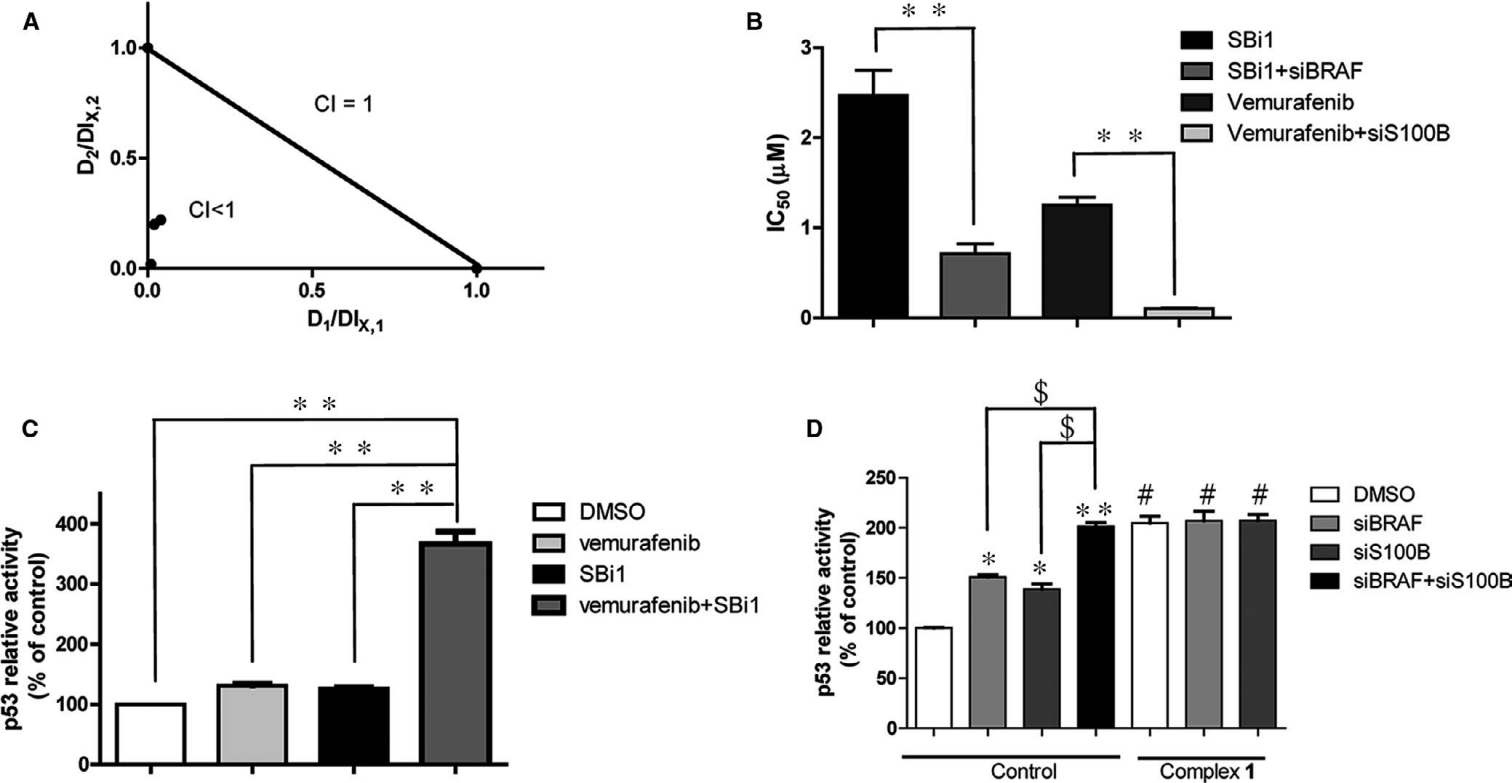
Cooperativity of S100B/p53 and MEK/ERK inhibition by complex **1**. (A) The combination index (CI) of co‐treatment of vemurafenib with SBi1. After co‐treatment of vemurafenib (2 μM) with SBi1 (0‐10 μM) in A375 cells for 72 h, cell viability was detected by the MTT assay, and then, CI values were calculated. D1 and D2 are the concentrations of vemurafenib and SBi1 used in the combination, and DL_X,1_ and DL_X,2_ are the concentrations of a single drug to produce the same effect. (B) The IC_50_ values of SBi1 and vemurafenib in knockdown A375 cells. S100B or BRAF knockdown cells were treated with SBi1 and vemurafenib, respectively, for 72 h, and cell viability was detected by the MTT assay. (C) p53 transcription activity in A375 cells. After treatment of vemurafenib (0.25 μM) together with SBi1 (0.25 μM) or alone (0.5 μM) for 12 h, p53 transcription activity was detected by the luciferase assay. (D) p53 transcription activity in A375 cells. After treatment with complex **1** for 12 h in BRAF and S100B knockdown cells, p53 transcription activity was detected by the luciferase assay. Error bars represent standard deviation of the means of the results from three independent experiments. *P < .05, **P < .01 compared with control group, ^$^ *P* < .05 compared with knockdown control, ^#^ *P* < .05 compared with corresponding control

RAF is an indirect negative regulator of p53, and inhibiting the RAF pathway would restore p53 transcription activity.^33^ However, a negative feedback loop exists whereby p53 up‐regulates S100B activity, thus reducing its own activity.^44^, ^45^ Thus, S100B might act as a redundant regulator to impede p53 activity when the BRAF/MEK/ERK pathway becomes blocked. We have already shown that complex **1** can block S100B and pan‐RAF pathway simultaneously via binding to BRAF, CRAF and S100B. However, the feedback loop that exists between S100B/p53 and BRAF pathway compelled us investigate whether complex **1** exerts a synergistic effect or additive effect when blocking these dual pathways. To investigate this, we initially evaluated the combination effect when simultaneously blocking the S100B and RAF/MEK/ERK pathway by their respective inhibitors. A combination index of < 0.3 was calculated from the cytotoxicity data after co‐treatment of the BRAF inhibitor vemurafenib (2 μM) with the S100B inhibitor SBi1 (0‐10 μM) in A375 cells (Figure 3A), indicating that there is a synergistic effect by blocking the S100B and BRAF pathways simultaneously.^46^ On the other hand, a combination index of > 0.8 was observed during co‐treatment of vemurafenib (2 μM) with the CRAF inhibitor sulindac sulfide (Figure S5E), indicating that there is an additive effect by blocking the CRAF and BRAF pathway simultaneously. To further verify the synergistic effects of dual inhibition of S100B and BRAF pathway simultaneously, we also investigated the effect of BRAF inhibition by vemurafenib on melanoma cytotoxicity in S100B knockdown A375 cells. The results showed that knockdown of S100B enhances cytotoxicity of vemurafenib to melanoma cells (Figure 3B). Similarly, abrogation of S100B activity by SBi1 enhanced cytotoxicity in BRAF knockdown A375 cells (Figure 3B). Taken together, these data indicate that blocking the S100B and BRAF pathways simultaneously might be a novel combinatorial therapeutic approach against melanoma. Studies have shown that both the BRAF and S100B pathway can regulate p53 transcription activity.^33^, ^44^, ^45^ To further investigate the mechanism of simultaneous inhibition of the S100B and BRAF/MEK/ERK pathways, the transcription activity of p53 was detected after co‐treatment with SBi1 and vemurafenib. The results revealed that co‐treatment of SBi1 (0.25 μM) with vemurafenib (0.25 μM) significantly increased p53 transcription activity, while a negligible effect was observed with SBi1 or vemurafenib alone at 0.5 μM (Figure 3C). This suggests that SBi1 and vemurafenib might exert a synergistic effect at inducing p53 transcription activity in melanoma cells, which could account for the synergistic cytotoxicity activity observed by dual S100B and BRAF inhibition in melanoma cells.

In light of the above findings, we hypothesized that complex **1** could block the S100B/p53 and RAF/MEK/ERK pathways simultaneously to produce a synergistic effect on the induction of p53 transcription activity. To investigate this, the transcription activity of p53 was detected after treatment with complex **1** in S100B or BRAF knockdown melanoma cells. The results showed that S100B or BRAF knockdown increased p53 transcription activity by 1.2‐fold and 1.4‐fold, respectively, while dual knockdown raised p53 transcription activity by around 2‐fold. Interestingly, treatment of complex **1** phenocopied the dual depletion of S100B and BRAF in terms of increasing p53 transcription activity in both control cells or single knockdown cells (Figure 3D). This result suggests that complex **1** can produce a synergistic effect to enhance p53 transcription activity via simultaneously blocking the S100B/p53 and RAF/MEK/ERK pathways in melanoma cells.

To further investigate the inhibition activity of complex **1** against other malignant cancer cell types, we measured the cytotoxicity of complex **1** against other cancer cell lines, such as lung cancer cells (A549 cells, BRAF‐wt), renal cancer cells (A498 cells, BRAF‐wt), colorectal cancer cells (HCT116, BRAF‐wt), and prostate cancer cells (DU145, p53‐mt). In order to confirm the important role of p53 against melanoma, we also tested the cytotoxicity of complex **1** against p53 knockdown A375 cells. The results showed that complex **1** displays weaker cytotoxicity in these cancer cell lines and is also less potent in p53 knockdown melanoma cells compared with A375 cells and A2058 cells (Figure S6). This suggests that the greater cytotoxicity of complex **1** against melanoma cells might be attributed to the potential target pathways of complex **1**, including the BRAF and p53 pathways, in melanoma.

### 3.5 Complex 1 suppresses malignant melanoma growth in vivo

**Figure 4:**
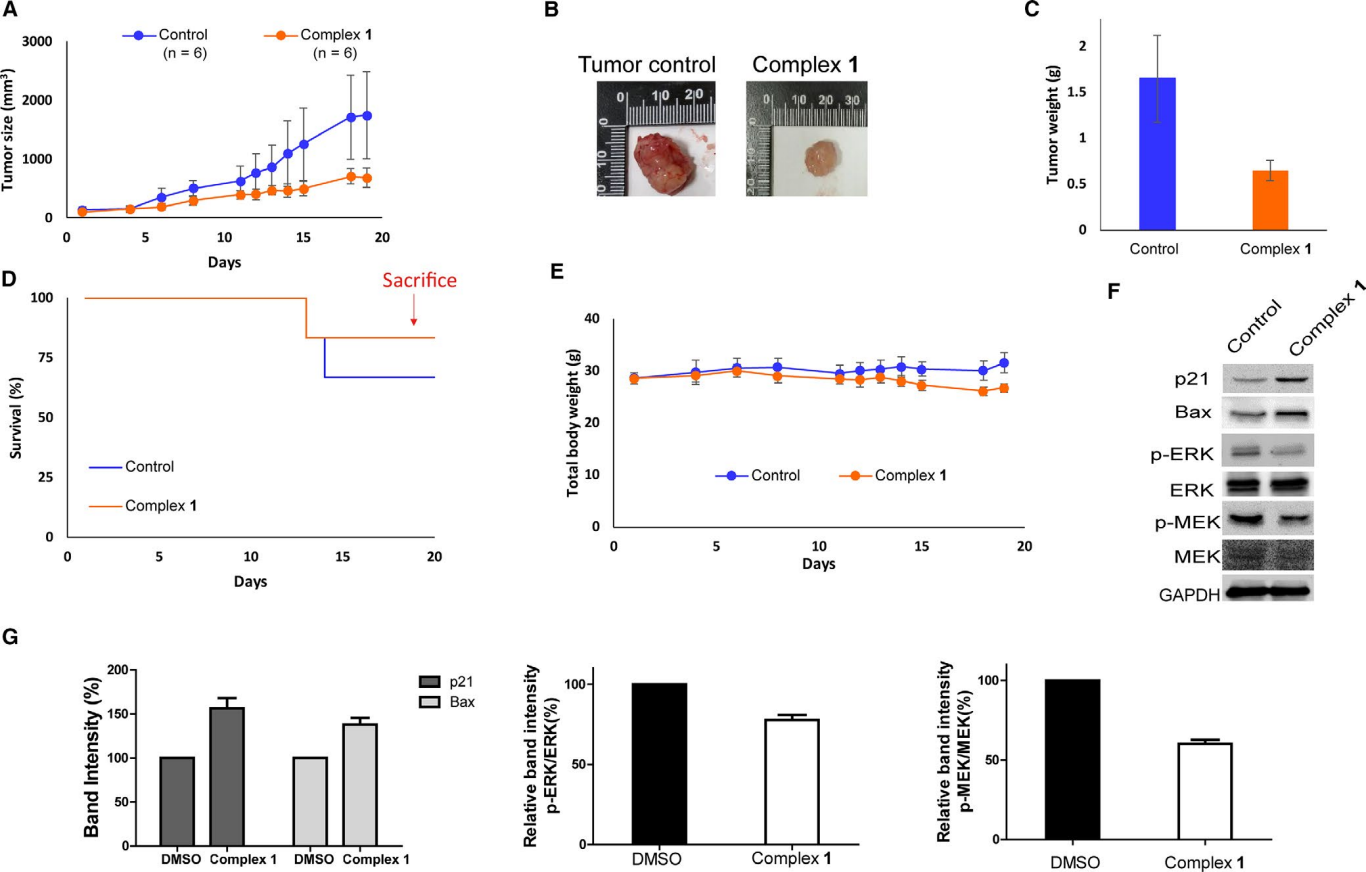
Complex **1** suppressed tumour growth in a A375 xenograft model in vivo and was well tolerated over the duration of treatment. (A) Effect of complex **1** on tumour size. (B) Representative photograph images of tumours in treatment and control groups. (C) Effect of complex **1** on tumour weight. (D) Effect of complex **1** on mouse total body weight. (E) Survival curves of mice in treatment and control groups. (F) Effect of complex **1** on p21, Bax, MEK, ERK, p‐MEK and p‐ERK protein levels in tumour tissues after sacrifice. (G) The protein band density in panel F was analysed. Error bars represent standard deviation of the means of the results from three independent experiments

As complex **1** displayed a promising anti‐proliferative activity against melanoma cells in vitro via targeting multiple pathways, we wished to investigate the viability of complex **1** to suppress melanoma tumour growth in vivo. Metal complexes normally display low solubility in aqueous solution, making them hard to administer orally, while subcutaneous administration is beneficial for drug delivery as the slow release profile of the complex prevents wasted drug.^47^, ^48^, ^49^ In the in vivo study, A375 cells were injected into NOD/SCID female mice to establish an A375 melanoma xenograft model. Then, complex **1** (1 mg/kg) was injected via subcutaneous injection twice a week for 20 days and tumour parameters were measured. Encouragingly, complex **1** significantly suppressed tumour volume (Figure 4A,B) and tumour mass (Figure 4C) by 2.6‐fold and 3.1‐fold respectively and extended the survival time of mice compared with the control group (Figure 4D). Importantly, complex **1** showed minimal effects on overall weight (Figure 4E), indicating that complex **1** can suppress the growth of malignant melanoma in vivo without observable toxicity. To further investigate the action of complex **1** in vivo, tumour specimens were examined after sacrifice. Complex **1** up‐regulated the expression of p21 and Bax as well as down‐regulated p‐MEK and p‐ERK at the protein level, suggesting that it could inhibit the MEK/ERK pathway and restore p53 function in vivo (Figure 4F,G). Taken together, these results indicate that complex **1** can extend survival time and suppress melanoma tumour growth in vivo, which can be attributed at least in part to its ability to simultaneously inhibit S100B/p53 together with the MEK/ERK pathway, without causing overt toxicity to the mice.

## 4 CONCLUSION

In summary, this study revealed that blocking BRAF and S100B pathway simultaneously might exert a synergistic effect to restore p53 function. Moreover, our study has identified complex **1** as a highly cytotoxic against malignant melanoma via blocking the S100B/p53 and pan‐RAF pathways. Complex **1** suppresses tumour growth in murine models of melanoma, leading to significantly improved survival time with no observable toxicity to mice. We envision that the blocking pan‐RAF and S100B/p53 pathways might be a novel synergistic effect strategy for melanoma therapy and that complex **1** is an attractive scaffold for the further development of anti‐melanoma drugs targeting the pan‐RAF and S100B pathways.

## CONFLICT OF INTEREST

The authors declare no conflict of interest.

## AUTHORS’ CONTRIBUTIONS

Ke‐Jia Wu, Shih‐Hsin Ho, Chun Wu, Hui‐Min David Wang, Dik‐Lung Ma and Chung‐Hang Leung: Conceptualization. Ke‐Jia Wu, Shih‐Hsin Ho and Chun Wu: Performing – experiments. Ke‐Jia Wu: Writing – original draft. All authors read and approved the final manuscript.

## Supporting information

Supplementary Material

## Data Availability

All data supporting the findings of this study are available within the article, within its supplementary information files and from the corresponding author upon reasonable request.

## References

[1] **Gray‐Schopfer** V , **Wellbrock** C , **Marais** R . Melanoma biology and new targeted therapy. Nature. 2007;445:851. 17314971 10.1038/nature05661

[2] Villanueva J , Vultur A , Lee JT , et al. Acquired resistance to BRAF inhibitors mediated by a RAF kinase switch in melanoma can be overcome by cotargeting MEK and IGF‐1R/PI3K. Cancer Cell. 2010;18:683‐695. 21156289 10.1016/j.ccr.2010.11.023 PMC3026446

[3] Montagut C , Sharma SV , Shioda T , et al. Elevated CRAF as a Potential Mechanism of Acquired Resistance to BRAF Inhibition in Melanoma. Cancer Res. 2008;68:4853‐4861. 18559533 10.1158/0008-5472.CAN-07-6787 PMC2692356

[4] Bittner M , Meltzer P , Chen Y , et al. Molecular classification of cutaneous malignant melanoma by gene expression profiling. Nature. 2000;406:536. 10952317 10.1038/35020115

[5] Cavalier MC , Pierce AD , Wilder PT , et al. Covalent small molecule inhibitors of ca2+‐bound s100b. Biochemistry. 2014;53:6628‐6640. 25268459 10.1021/bi5005552 PMC4211652

[6] Robert C , Thomas L , Bondarenko I , et al. Ipilimumab plus dacarbazine for previously untreated metastatic melanoma. N Engl J Med. 2011;364:2517‐2526. 21639810 10.1056/NEJMoa1104621

[7] Doudican N , Orlow S . Inhibition of the CRAF/prohibitin interaction reverses CRAF‐dependent resistance to vemurafenib. Oncogene. 2017;36:423. 27321184 10.1038/onc.2016.214

[8] Karasarides M , Chiloeches A , Hayward R , et al. B‐RAF is a therapeutic target in melanoma. Oncogene. 2004;23:6292. 15208680 10.1038/sj.onc.1207785

[9] Ehrenreiter K , Kern F , Velamoor V , et al. Raf‐1 addiction in Ras‐induced skin carcinogenesis. Cancer Cell. 2009;16:149‐160. 19647225 10.1016/j.ccr.2009.06.008

[10] Gollob JA , Wilhelm S , Carter C , Kelley SL . Role of Raf kinase in cancer: therapeutic potential of targeting the Raf/MEK/ERK signal transduction pathway. Semin Oncol. 2006;33(4):392‐406. 16890795 10.1053/j.seminoncol.2006.04.002

[11] Rebocho AP , Marais R . New insight puts CRAF in sight as a therapeutic target. Cancer Disc. 2011;1:98‐99. 10.1158/2159-8290.CD-11-0118 PMC3272431 22318779

[12] Montagut C , Settleman J . Targeting the RAF–MEK–ERK pathway in cancer therapy. Cancer Lett. 2009;283:125‐134. 19217204 10.1016/j.canlet.2009.01.022

[13] Dumaz N . Mechanism of RAF isoform switching induced by oncogenic RAS in melanoma. Small GTPases. 2011;2:584‐591. 10.4161/sgtp.2.5.17814 PMC3265821 22292133

[14] Kang J , Nam JS , Lee HJ , et al. Chemical strategies to modify amyloidogenic peptides by iridium (III) complexes: Coordination and photo‐induced oxidation. Chem Sci. 2019;10(28):6855‐6862. 31391908 10.1039/c9sc00931k PMC6657414

[15] Liu Z , Li J , Ge X , Zhang S , Xu Z , Gao W . Design, synthesis, and evaluation of phosphorescent Ir (III) complexes with anticancer activity. J Inorg Biochem. 2019;197:110703. 31077890 10.1016/j.jinorgbio.2019.110703

[16] Ma D‐L , Chan DS‐H , Leung C‐H . Group 9 organometallic compounds for therapeutic and bioanalytical applications. Acc Chem Res. 2014;47:3614‐3631. 25369127 10.1021/ar500310z

[17] Guan R , Chen Y , Zeng L , et al. Oncosis‐inducing cyclometalated iridium (iii) complexes. Chem Sci. 2018;9:5183‐5190. 29997872 10.1039/c8sc01142g PMC6000986

[18] Du F , Bai L , He M , et al. Design, synthesis and biological evaluation of iridium (III) complexes as potential antitumor agents. J Inorg Biochem. 2019;201:110822. 31536949 10.1016/j.jinorgbio.2019.110822

[19] Ma W , Ge X , Guo L , et al. Bichromophoric anticancer drug: Targeting lysosome with rhodamine modified cyclometalated Iridium (III) complexes. Dyes Pigm. 2019;162:385‐393.

[20] Ma D‐L , Ma VP‐Y , Chan DS‐H , Leung K‐H , He H‐Z , Leung C‐H . Recent advances in luminescent heavy metal complexes for sensing. Coord Chem Rev. 2012;256:3087‐3113.

[21] Xiao Z , Jiang R , Jin J , et al. Diiron (II) pentacarbonyl complexes as CO‐releasing molecules: their synthesis, characterization, CO‐releasing behaviour and biocompatibility. Dalton Trans. 2019;48:468‐477. 30488059 10.1039/c8dt03982h

[22] Liu L‐J , Wang W , Huang S‐Y , et al. Inhibition of the Ras/Raf interaction and repression of renal cancer xenografts in vivo by an enantiomeric iridium (III) metal‐based compound. Chem Sci. 2017;8:4756‐4763. 28959398 10.1039/c7sc00311k PMC5603957

[23] Blazer LL , Roman DL , Muxlow MR , Neubig RR . Use of Flow Cytometric Methods to Quantify Protein‐Protein Interactions. Curr Protoc Cytom. 2010;51(13):pp. 1. 1‐.1. 5. 10.1002/0471142956.cy1311s51 PMC2849137 20069525

[24] Kilpin KJ , Dyson PJ . Enzyme inhibition by metal complexes: concepts, strategies and applications. Chem Sci. 2013;4:1410‐1419.

[25] Kang T‐S , Ko C‐N , Zhang J‐T , et al. Rhodium (III)‐Based Inhibitor of the JMJD3‐H3K27me3 Interaction and Modulator of the Inflammatory Response. Inorg Chem. 2018;57:14023‐14026. 30375229 10.1021/acs.inorgchem.8b02256

[26] Santarpia L , Lippman SM , El‐Naggar AK . Targeting the MAPK–RAS–RAF signaling pathway in cancer therapy. Expert Opin Ther. 2012;16:103‐119. 10.1517/14728222.2011.645805 PMC3457779 22239440

[27] Kwong LN , Chin L . The brothers RAF. Cell. 2010;140:180‐182. 20141832 10.1016/j.cell.2010.01.013

[28] Uht RM , Amos S , Martin PM , Riggan A , Hussaini IM . The protein kinase C‐η isoform induces proliferation in glioblastoma cell lines through an ERK/Elk‐1 pathway. Oncogene. 2007;26:2885. 17146445 10.1038/sj.onc.1210090

[29] Wellbrock C , Karasarides M , Marais R . The RAF proteins take centre stage. Nat Rev Mol Cell Biol. 2004;5:875. 15520807 10.1038/nrm1498

[30] Cohen C , Zavala‐Pompa A , Sequeira JH , et al. Mitogen‐actived protein kinase activation is an early event in melanoma progression. Clin Cancer Res. 2002;8:3728‐3733. 12473582

[31] Jafari R , Almqvist H , Axelsson H , et al. The cellular thermal shift assay for evaluating drug target interactions in cells. Nat Protoc. 2014;9:2100. 25101824 10.1038/nprot.2014.138

[32] Molina DM , Jafari R , Ignatushchenko M , et al. Monitoring drug target engagement in cells and tissues using the cellular thermal shift assay. Science. 2013;341:84‐87. 23828940 10.1126/science.1233606

[33] Drosten M , Sum EY , Lechuga CG , et al. Loss of p53 induces cell proliferation via Ras‐independent activation of the Raf/Mek/Erk signaling pathway. Proc Natl Acad Sci USA. 2014;111:15155‐15160. 25288756 10.1073/pnas.1417549111 PMC4210339

[34] Salic A , Mitchison TJ . A chemical method for fast and sensitive detection of DNA synthesis in vivo. Proc Natl Acad Sci USA. 2008;105:2415‐2420. 18272492 10.1073/pnas.0712168105 PMC2268151

[35] Donato R , Sorci G , Riuzzi F , et al. S100B's double life: intracellular regulator and extracellular signal. Biochimica et BBA‐Mol Cell Res. 2009;1793:1008‐1022. 10.1016/j.bbamcr.2008.11.009 19110011

[36] Lin J , Blake M , Tang C , et al. Inhibition of p53 transcriptional activity by the S100B calcium‐binding protein. J Biol Chem. 2001;276:35037‐35041. 11454863 10.1074/jbc.M104379200

[37] Baudier J , Delphin C , Grunwald D , Khochbin S , Lawrence JJ . Characterization of the tumor suppressor protein p53 as a protein kinase C substrate and a S100b‐binding protein. Proc Natl Acad Sci USA. 1992;89:11627‐11631. 1454855 10.1073/pnas.89.23.11627 PMC50606

[38] Djukanovic D , Hofmann U , Sucker A , Rittgen W , Schadendorf D . Comparison of S100 protein and MIA protein as serum marker for malignant melanoma. Anticancer Res. 2000;20:2203‐2207. 10928178

[39] Wu J‐X , Zhang D‐G , Zheng J‐N , Pei D‐S . Rap2a is a novel target gene of p53 and regulates cancer cell migration and invasion. Cell Signal. 2015;27:1198‐1207. 25728512 10.1016/j.cellsig.2015.02.026

[40] Capoccia E , Cirillo C , Marchetto A , et al. S100B–p53 disengagement by pentamidine promotes apoptosis and inhibits cellular migration via aquaporin‐4 and metalloproteinase‐2 inhibition in C6 glioma cells. Oncol Lett. 2015;9:2864‐2870. 26137161 10.3892/ol.2015.3091 PMC4473713

[41] Wu K‐J , Ho S‐H , Dong J‐Y , et al. Aliphatic Group‐Tethered Iridium Complex as a Theranostic Agent against Malignant Melanoma Metastasis. ACS Appl Bio Mater. 2020;3:2017‐2027. 10.1021/acsabm.9b01156 35025323

[42] Fernandez‐Fernandez MR , Rutherford TJ , Fersht AR . Members of the S100 family bind p53 in two distinct ways. Protein Sci. 2008;17:1663‐1670. 18694925 10.1110/ps.035527.108 PMC2548378

[43] Aubrey BJ , Kelly GL , Janic A , Herold MJ , Strasser A . How does p53 induce apoptosis and how does this relate to p53‐mediated tumour suppression? Cell Death Differ. 2018;25:104‐113. 29149101 10.1038/cdd.2017.169 PMC5729529

[44] Wilder PT , Lin J , Bair CL , et al. Recognition of the tumor suppressor protein p53 and other protein targets by the calcium‐binding protein S100B. Biochimica et BBA‐Mol Cell Res. 2006;1763:1284‐1297. 10.1016/j.bbamcr.2006.08.024 17010455

[45] Lin J , Yang Q , Yan Z , et al. Inhibiting S100B restores p53 levels in primary malignant melanoma cancer cells. J Biol Chem. 2004;279:34071‐34077. 15178678 10.1074/jbc.M405419200

[46] Lehár J , Krueger AS , Avery W , et al. Synergistic drug combinations tend to improve therapeutically relevant selectivity. Nat Biotechnol. 2009;27:659. 19581876 10.1038/nbt.1549 PMC2708317

[47] Petrilli R , Eloy JO , Saggioro FP , et al. Skin cancer treatment effectiveness is improved by iontophoresis of EGFR‐targeted liposomes containing 5‐FU compared with subcutaneous injection. J Control Release. 2018;283:151‐162. 29864476 10.1016/j.jconrel.2018.05.038

[48] Kaur G , Willsmore T , Gulati K , et al. Titanium wire implants with nanotube arrays: a study model for localized cancer treatment. Biomaterials. 2016;101:176‐188. 27289379 10.1016/j.biomaterials.2016.05.048

[49] Zhou EY , Knox HJ , Reinhardt CJ , Partipilo G , Nilges MJ , Chan J . Near‐infrared photoactivatable nitric oxide donors with integrated photoacoustic monitoring. J Am Chem Soc. 2018;140:11686‐11697. 30198716 10.1021/jacs.8b05514 PMC7331458

