# Supplementary material for: Simultaneous blocking of the pan‐RAF and S100B pathways as a synergistic therapeutic strategy against malignant melanoma
Source: J Cell Mol Med. 2020 Dec 30;25(4):1972–81. doi: 10.1111/jcmm.15994 (PMC7882986; doi:10.1111/jcmm.15994)
Supplement: Supplementary file 1 — Supplementary Material [file JCMM-25-1972-s001.docx]

Supporting Information

**Simultaneous blocking of the pan-RAF and S100B pathways as a synergistic therapeutic strategy against malignant melanoma**

Ke-Jia Wu^+[1]^, Shih-Hsin Ho^+[2]^, Chun Wu^+[3]^,  Hui-Min David Wang*^[4,5,6]^, Dik-Lung Ma*^[3]^ and Chung-Hang Leung*^[1]^

^1^ State Key Laboratory of Quality Research in Chinese Medicine, Institute of Chinese Medical Sciences, University of Macau, Macao SAR, 999078, P. R. China.

^2^ State Key Laboratory of Urban Water Resource and Environment, School of Environment, Harbin Institute of Technology,

Harbin, 150090, P. R. China

^3^ Department of Chemistry, Hong Kong Baptist University, Kowloon Tong, Hong Kong, 999077, P. R China

^4^ Graduate Institute of Biomedical Engineering National Chung Hsing University, Taichung, 402, Taiwan

^5^ Graduate Institute of Medicine, College of Medicine, Kaohsiung Medical University, Kaohsiung, 807, Taiwan

^6^ Department of Medical Laboratory Science and Biotechnology, China Medical University, Taichung City, 404, Taiwan

+ These authors contributed equally to this work

**Methods**

**General experimental.** Mass spectrometry was performed at the Mass Spectroscopy Unit at the Department of Chemistry, Hong Kong Baptist University, Hong Kong (China). Deuterated solvents for NMR purposes were obtained from Armar and used as received.^1^H and ^13^C NMR were recorded on a Bruker Advance 400 spectrometer operating at 400 MHz (^1^H) and 100 MHz (^13^C). ^1^H and ^13^C chemical shifts were referenced internally to solvent shift (Acetonitrile-d_3_: ^1^H, δ1.94, ^13^Cδ118.7; Acetone-d_6_: ^1^H δ2.05, ^13^Cδ29.7). Chemical shifts (d) are quoted in ppm, the downfield direction being defined as positive. Uncertainties in chemical shifts are typically ±0.01 ppm for ^1^H and ±0.05 for ^13^C. Coupling constants are typically ± 0.1 Hz for ^1^H-^1^H and ±0.5 Hz for ^1^H-^13^C couplings. The following abbreviations are used for convenience in reporting the multiplicity of NMR resonances: s, singlet; d, doublet; t, triplet; q, quartet; m, multiplet. All NMR data was acquired and processed using standard Bruker software (Topspin).

**Chemicals and reagents**: Dual-Luciferase^®^ Reporter Assay System was purchased from (Promega, Madison, WI, USA). p53-Luciferase plasmid was purchased from Beyotime (Beyotime, Shanghai, China). Iridium chloride hydrate (IrCl_3_·xH2O) and rhodium chloride hydrate (RhCl_3_·xH2O) were purchased from Precious Metals Online (Australia). The synthesis and stability experiments of compounds were described in SI Materials and Methods. Transfection Reagent was obtained from Thermo Scientific (Thermo Scientific, St Leon-Rot, Germany). p53 antibody, S100B antibody were purchased from Abcam (Abcam Inc., Cambridge, MA, USA), p21 antibody, Cyclin D1 antibody, MEK antibody, phosphor-MEK antibody, ERK antibody, phosphor-ERK antibody, and GAPDH antibody were purchased from CST (CST Inc., Massachusetts, USA). S100B protein was purchased from R&D Systems (R&D Systems Inc., Minneapolis, Minnesota, USA). p53 peptide was purchased from GL Biochem Ltd. (GL Biochem Ltd., Shanghai, China). All the compounds were dissolved in dimethyl sulfoxide (DMSO). Human malignant melanoma (A375 cells, A2058 cells) were obtained from American Type Culture Collection. The hepatocyte cells (LO2 cells) was obtained from Chinese Academy of Science (Cell Biology of Shanghai Institute, Shanghai, China).

**Synthesis of rhodium(III) and iridium(III) complexes**

**Complex 1**: Reported.^[^[^1^](#_ENREF_1)^]^

**Complex 2**: Reported.^[^[^1^](#_ENREF_1)^]^

**Complex 3**: Reported.^[^[^1^](#_ENREF_1)^]^

**Complex 4**: Reported.^[^[^1^](#_ENREF_1)^]^

**Complex 5:** Reported.^[^[^1^](#_ENREF_1)^]^

**Complex 6:** Reported.^[^[^1^](#_ENREF_1)^]^

**Complex 7:** Reported.^[^[^1^](#_ENREF_1)^]^

**CI**: Reported.^[^[^1^](#_ENREF_1)^]^

**UV/Vis absorbance spectroscopy**: Complex **1** (5 μM) was added to 0, 0.01, 0.1, 1, 10 mM of Ca^2+^ in 20 mM Tris-HCl buffer (pH = 7.4) at 298 K. Absorption spectra were recorded on Cary UV-100 Spectrophotometer at a range of 200 nm to 800 nm. The absorbance was corrected by subtraction of 20 mM Tris-HCl buffer (pH = 7.4) as the background absorbance.

**Stability experiments**. For ^1^H NMR, complex **1** (5 mM) was dissolved in 90% [*d_6_*]DMSO/10% D_2_O at 298 K over 7 days. ^1^H NMR measurements were carried out on a 400 MHz Bruker instrument. For UV-Visible spectrometry, complex **1** (5 μM) was dissolved in 80% acetonitrile/20% 20 mM Tris-HCl buffer (pH = 7.4) at 298 K over 7 days. Absorption spectra were recorded on a Cary UV-100 Spectrophotometer at a range of 200 nm to 800 nm. The absorbance of complex **1** was corrected by subtraction of 80% acetonitrile/20% 20 mM Tris-HCl buffer (pH = 7.4) as the background absorbance.

**Cell cultures**: The cells were cultivated in DMEM medium with 1% penicillin (100 units/mL)/streptomycin (100 μg/mL) and 10% fetal bovine serum (FBS). Cells were maintained at a density of 6 × 10^5^ cell/mL in 5% CO_2_ at 37 °C.

**Flow cytometry protein interaction assay**. The EZ-Link Sulfo-NHS-LC-Biotinylation Kit was used to conjugate biotin and S100B protein to polystyrene beads in Ca^2+^ buffer. The procedure was performed as described.^[^[^2^](#_ENREF_2)^]^ After conjugating S100B protein to the beads, human p53 peptide labeled with FITC was added, and FITC fluorescence was detected by flow cytometry**.**

**Co-IP assay**: The co-IP assay was performed as previously described.^[^[^3^](#_ENREF_3)^]^ Briefly, A375 cells were seeded at a density of 2 × 10^6^ cells in a six-well plate. Cells were treated with the 0.1 and 1.0 μM complex **1** or 10 μM SBi1 for 12 h. Cells were lysed and protein samples were collected. The concentration of protein samples was calculated using the Pierce BCA protein assay kit. 30 μg of each protein sample were incubated overnight with 10 μL pre-incubated anti-p53 magnetic beads according to the manufacturer's protocol. The complex was washed 5 times to elute non-specific and non-cross-linked antibodies. Then, the precipitated proteins were subjected to SDS-PAGE and analyzed by Western blotting with the indicated antibodies.

**Western blotting**: A375 cells were seeded at a density of 6 × 10^5^ cell in a 6-well plate overnight. Cells were treated with complex **1** (in 0.1% DMSO), SBi1, or vehicle control in 1% FBS medium for an additional 12 h. Cells were lysed, and protein samples were collected. Western blotting analysis was performed as described.^[^[^4^](#_ENREF_5)^]^

**Transient transfection**: A375 cells were seeded in a culture dish overnight before transfection. p53-luciferase plasmid, and TurboFect reagent were mixed together in Dulbecco's modified eagle medium (DMEM) without FBS and the resulting solution was incubated for 20 min at 37 °C. The mixture was then added to the A375 cells in the wells. The cells were incubated for 12 h at 37 °C in a CO_2_ incubator before use.

**Luciferase reporter assay**: The inhibition of p53 activity was assayed by a luciferase reporter assay system (Promega, Madison, WI, USA). Briefly, A375 cells were seed at a density of 6 × 10^5^ cell in a culture dish overnight. The cells were co-transfected with p53-luciferase plasmid in serum-free DMEM medium using TurboFect Transfection Reagent. Then, the transfected cells were seeded in a 24-well plate and treated with an indicated concentration of 1 in 1% FBS medium for 6h. The transfected cells were lysed by the addition of 160 μL Passive Lysis Buffer (PLB). 50 μL of the cell lysates were transferred to a 96-well white plate followed by adding 50 μL of luciferase reporter reagent (LAR). The transcriptional activity was determined by measuring the activity of firefly luciferase in SpectraMax M5 microplate reader (Molecular Devices).

**Inductively coupled plasma mass spectrometry (ICP-MS):** 500 μL of the cell samples from nuclear extraction or whole cell lysate was then combined with 1500 μL of a 68% HNO_3_: H_2_O_2_ (v/v = 4:1) solution, while the remainder of the lysate was quantified for protein by a bicinchoninic assay (BCA). The 2% HNO_3_ solution was analyzed for iridium uptake levels on a Thermo iCAP Qs ICP-MS. Iridium uptake levels in each sample were normalized to the concentration of corresponding protein and calculated each value of ng [Iridium]/mg [protein].

**Knockdown assay**: A375 cells were seeded in 6-well plate at 80% confluences in DMEM medium for 24 h. Lipo3000 reagent and S100B siRNA (5′‐GAA CAU GAG UGA GAU UAG ATT‐3′ (sense), 5′‐UCU AAU CUC ACU CAU GUU CTT‐3′ (antisense)) was gently mixed and incubated for 20 min at room temperature. Then, 500 µL of the Lipo3000/siRNA mixture were added to each well. Cells were incubated at 37 °C in a CO_2_ incubator for 48 h post-transfection before further research.

**Cellular thermal shift assay**: Cellular thermal shift assay was performed to monitor the target engagement of **1** in A375 cell lysates. Briefly, cell lysates from 2 × 10^6^ A375 cells were collected, diluted in PBS and separated in the same aliquots. Each aliquot was treated with **1** (10.0 μM) or DMSO 30 min after incubation at room temperature, the compound-treated lysates were divided into 50 μL in each of PCR tubes and heated individually at different temperatures (Veriti thermal cycler, Applied Biosystems/Life Technologies). The heated lysates were centrifuged and the supernatants were analyzed by SDS-PAGE followed by immunoblotting analysis by probing with the indicated antibody.

**p53 degradation assay:** After treatment with complex **1** for 12 h, A375 cells were treated with 50 μg/ml cycloheximide (CHX, Sigma-Aldrich) for the indicated time periods. p53 levels were determined by Western blot analysis and quantified by densitometry analysis.

**Apoptosis assay:** An FITC-Annexin apoptosis detection kit (BD Biosciences, San Jose, CA, USA) was utilized. The experiment was performed according to the manufacturer’s instructions. Briefly, A375 cells were seeded at density of 1 × 10^6^ in a 6-well plate, and were treated with **1** (0.1-1 μM) for 12 h. Cells were harvested and washed twice with ice-cold PBS, and then resuspended in 1 × binding buffer followed by incubation with Annexin V/PI solution for 15 min at room temperature. The samples were immediately analyzed by flow cytometry using a BD Biosciences BD LSRFortessa™ Flow Cytometer (BD Biosciences). At least 1 × 10^5^ cells were analyzed for each sample.

**MTT assay**: A375 and LO2 cells were seeded at 5000 cells per well in a 96-well plates and incubated overnight at 37 °C. The cells were treated with **1** at final concentration from 0.01 to 10 μM for 72 h. Then 3-(4,5-[di](https://en.wikipedia.org/wiki/Di-)[methyl](https://en.wikipedia.org/wiki/Methyl)[thiazol](https://en.wikipedia.org/wiki/Thiazole)-2-yl)-2,5-di[phenyl](https://en.wikipedia.org/wiki/Phenyl)tetrazolium bromide (MTT) reagent was added to each well at a final concentration of 0.5 mg/mL for a further 4 h. After then, the medium was replaced with 100 μL DMSO. The viability of the cells was measured by recording the absorbance of each well at 490 nm using a SpectraMax M5 microplate reader after shaking the plate for 10 min at room temperature in the dark.

**Melanoma xenograft assay**: NOD.CB17-Prkdcscid / NcrCrl (NOD / SCID) female mice were purchased from BioLASCO Experimental Animal Center (Taiwan Co., Ltd)., and injected with A375 cells at 8 weeks of age. The injection site was sanitized using 70% ethyl alcohol. 1 × 10^6^ A375 cells dissolved in 0.1 mL of PBS were subcutaneously implanted into mice by using a 27-gauge (G) needle. The mice were observed for 7 to 10 days, until the tumor grew to an appropriate size (about 100 mm^3^). The testing drug was administrated via subcutaneous injection to mice and observe its therapeutic effect on melanoma tumors ^[^[^1^](#_ENREF_1)^]^.

**Sample preparation from animal tissues**: Mouse tumor tissues were chopped and rinsed in ice-cold saline solution. The tissue was immersed with 10 times the volume of RIPA, and then homogenized with a Mixer Mill MM 300 homogenizer (Qiagen, Chatsworth, CA). The mixture was further incubated with RIPA for 10 min on ice and then centrifuged at 12000 g for 30 min, and the supernatant was collected and analyzed by Western blotting.

**Statistical analysis**: For statistical analysis, all data were analyzed with one-way analysis of variance (ANOVA) followed by the Dunnett's method for multiple comparisons by using GraphPad Prism 5.0.

**References**

[1] L.-J. Liu, W. Wang, S.-Y. Huang, Y. Hong, G. Li, S. Lin, J. Tian, Z. Cai, H.-M. D. Wang, D.-L. Ma, *Chemical science* **2017**, *8*, 4756-4763.

[2] L. L. Blazer, D. L. Roman, M. R. Muxlow, R. R. Neubig, *Current protocols in cytometry* **2010**, *51*, 13.11. 11-13.11. 15.

[3] aG. M. Bol, F. Vesuna, M. Xie, J. Zeng, K. Aziz, N. Gandhi, A. Levine, A. Irving, D. Korz, S. Tantravedi, M. R. H. Van Voss, K. Gabrielson, E. A. Bordt, B. M. Polster, L. Cope, P. van der Groep, A. Kondaskar, M. A. Rudek, R. S. Hosmane, E. van der Wall, P. J. van Diest, P. T. Tran, V. Raman, *EMBO Mol. Med.* **2015**, *7*, 648-669; bC. Yang, W. Wang, L. Chen, J. Liang, S. Lin, M. Y. Lee, D. L. Ma, C. H. Leung, *Chem. Commun. (Camb.)* **2016**, *52*, 12837-12840.

[4] C. Yang, W. Wang, G. D. Li, H. J. Zhong, Z. Z. Dong, C. Y. Wong, D. W. Kwong, D. L. Ma, C. H. Leung, *Sci Rep* **2017**, *7*, 42860.

**Supplementary Tables**

## Table S1. Primer sequences used in this paper.

| Name | sense | antisense |
| --- | --- | --- |
| p21 | 5′-CAGAGGCGGAGGAGAACAAA-3′ | 5′-ATGGAGGGCGGATTGGAA-3′ |
| Bax | 5′-TGTTTTCTGACGGCAACTTCA-3′ | 5′-AGCCCATGATGGTTCTGATCA-3′ |

#

#

Supplementary Figures

FIGURE S1**.** Chemical structures of complexes **1**-**7** and CI.


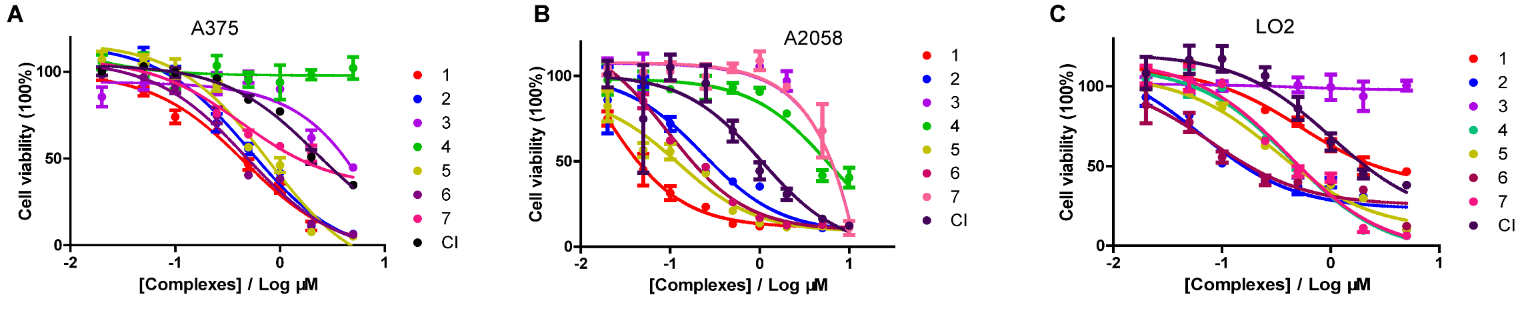
FIGURE S2. Raw cytotoxicity data of complex **1** in different cell lines. (A) A375 cell, (B) A2058 cell, (C) LO2 cell.


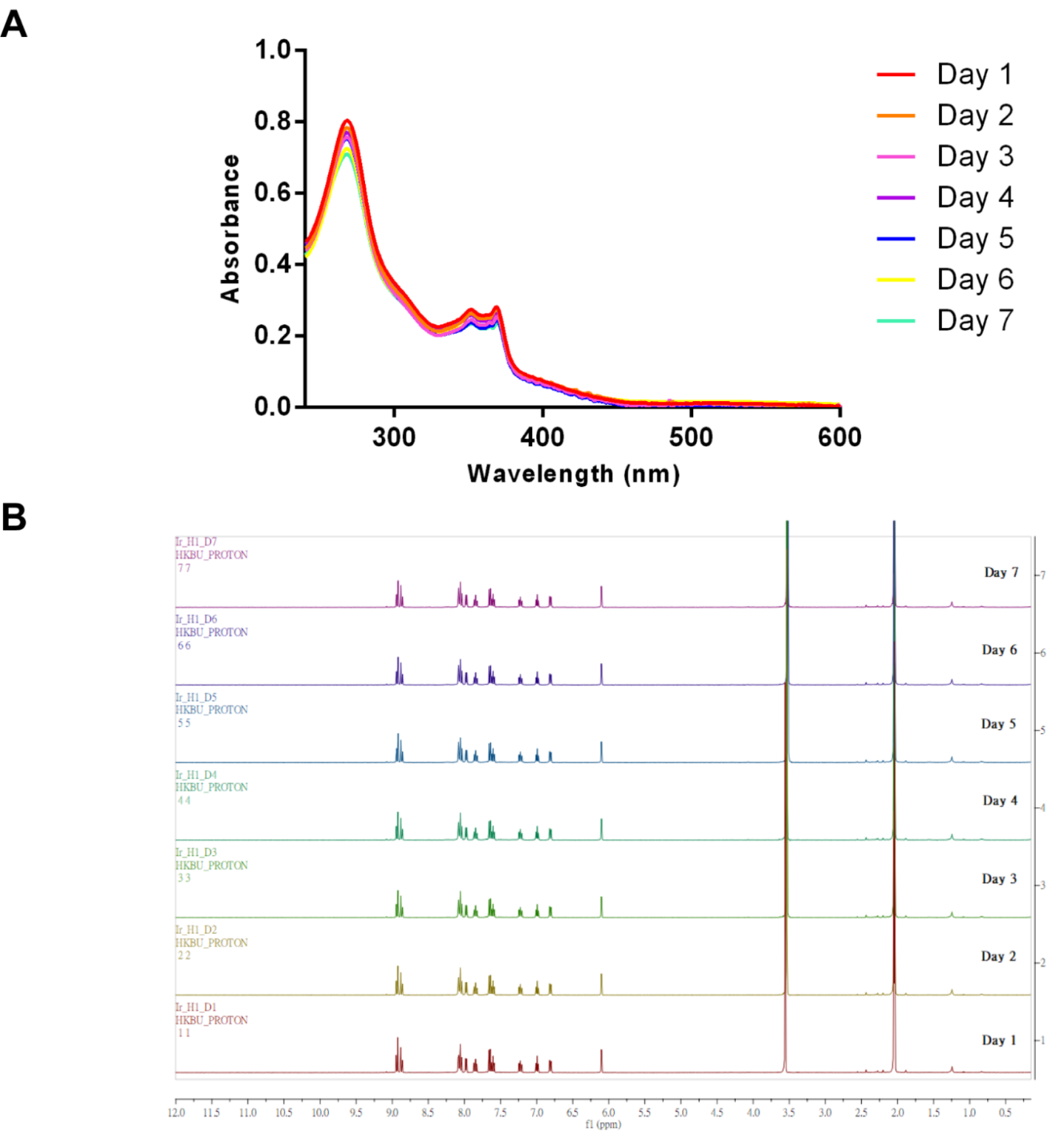


FIGURE S3. Stability of complex **1**. (A) UV-Vis absorption of complex **1** at 5 μM in 80% acetonitrile/20% 20 mM Tris-HCl buffer (pH=7.4) at 298 K over 7 days. (B) ^1^H NMR spectra of complex **1** at 5 mM in 90%[*d_6_*]DMSO/10% D_2_O at 298 K over 7 days.


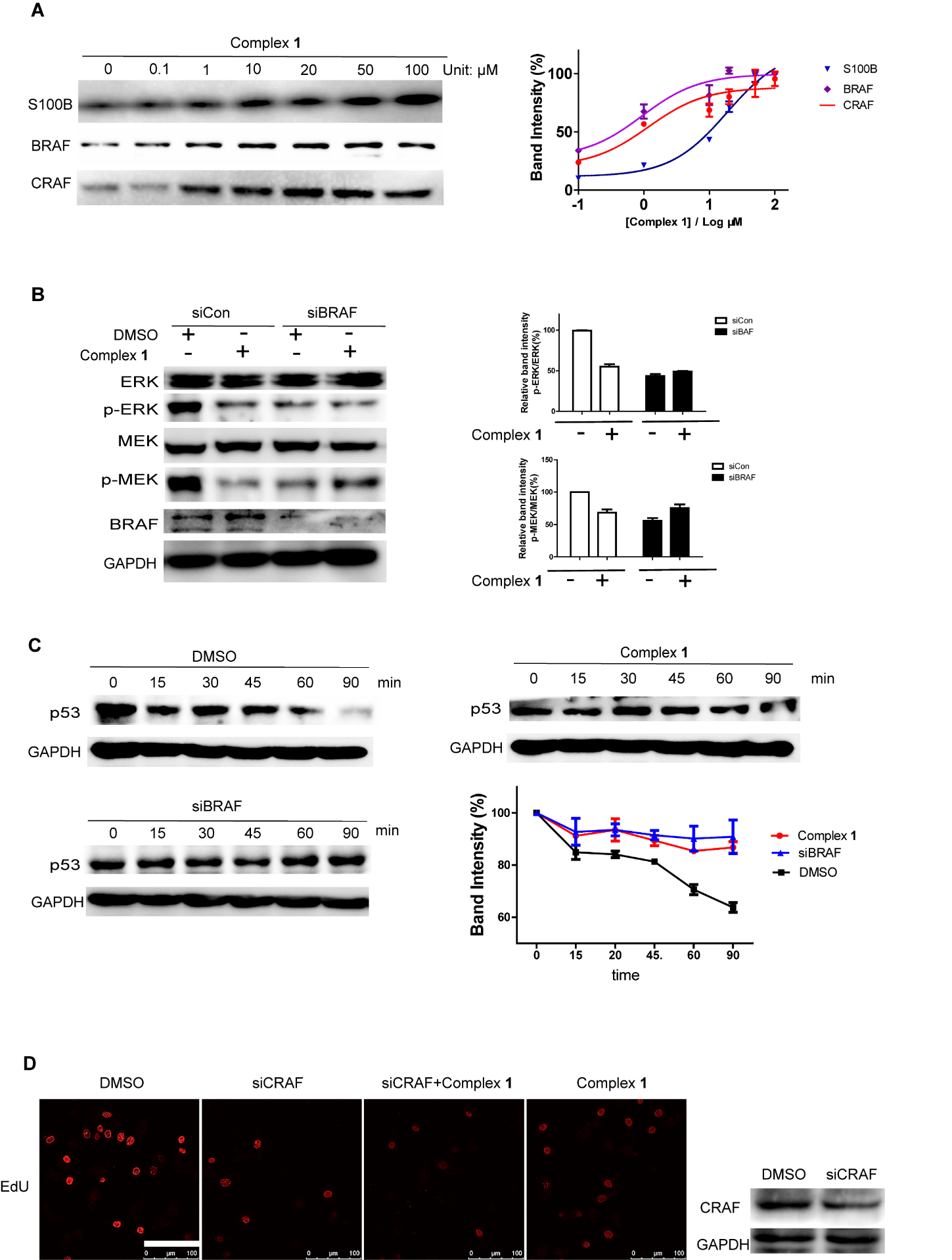
FIGURE S4. (A) ITDRF_CETSA_ curves of S100B, BRAF and CRAF protein in the presence of complex **1** (0-100 μM). (B) The effect of complex **1** on ERK, p-ERK, MEK and p-MEK protein expression after BRAF knockdown by BRAF siRNA in A375 cells.The protein samples were detected by Western blotting and the band density were analyzed. (C) A375 cells or BRAF knockdown A375 cells were treated with compound **1** (1 µM) for 12 h, and then the cells were treated with cycloheximide (CHX) for 0, 15, 30, 60 and 90 min. Equal amounts of whole cell lysates were analyzed by Western blot with a p53 antibody. GAPDH was used as an internal control. The protein samples were detected by Western blotting and the band density were analyzed. (D) Confocal image of A375 cells and knockdown CRAF A375 cells after treatment with complex **1** and staining for EdU.

**
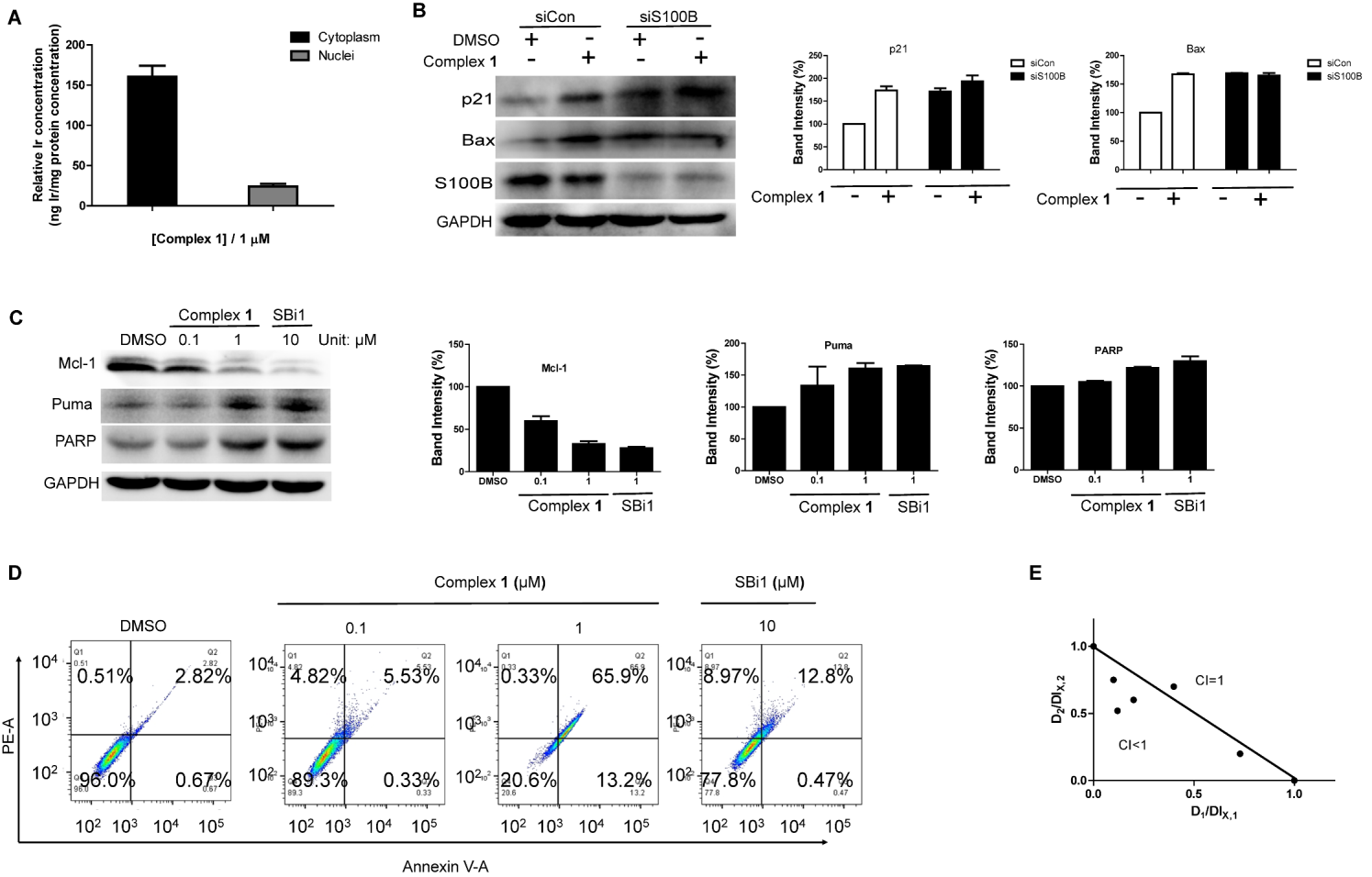
**

FIGURE S5. The mechanism of complex **1**. (A) Iridium accumulation in nuclear and cytoplasmic lysates by ICP-MS. A375 cells were treated with complex **1** (1 μM) for 12 h before harvesting. (B) The effect of complex **1** on p21, Bax protein expression after S100B knockdown by S100B siRNA in A375 cells. Protein samples were analyzed by Western blotting and the band density was calculated. (C) The effect of complex **1** on Mcl-1, Puma, PARP protein expression after treated with complex **1** for 12h in A375 cells. The protein samples were detected by Western blotting and the band density were analyzed. (D) The effect of complex **1** on apoptosis was evaluated by flow cytometry assay. A375 cells were treated with complex **1** (0.1-1 μM) for 12 h before harvesting. At least 1 × 10^5^ cells were analyzed for each sample. (E) The combination index of co-treatment of sulindac sulfide with vemurafenib. After co-treatment of sulindac sulfide (0-50 μM) with vemurafenib (2 μM) for 72 h, cell viability was detected by the MTT assay. The combination index was calculated.

**
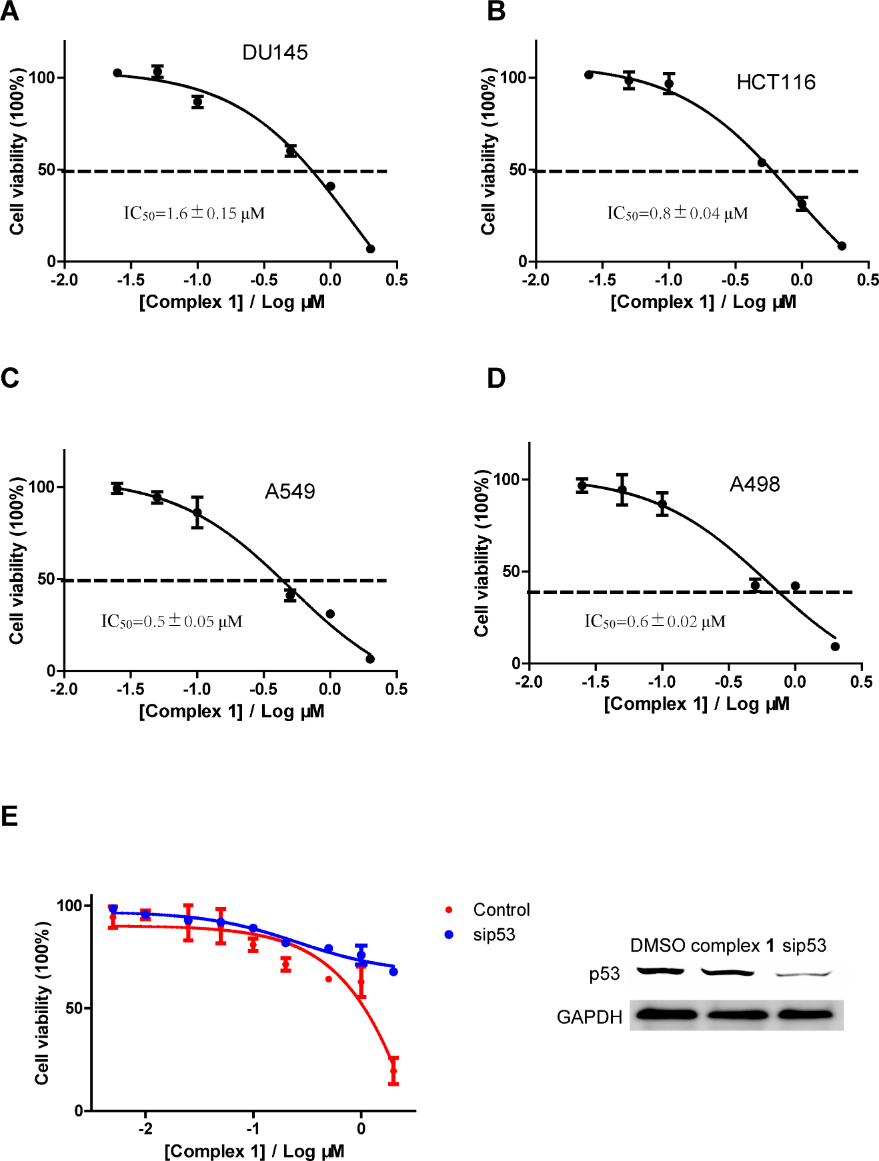
**FIGURE S6. The cytotoxicity of complex **1** in different cells. After treatment with complex **1** for 72 h, cell viability was detected by the MTT assay. (A) DU145 cell viability. (B) HCT116 cell viability. (C) A549 cell viability. (D) A498 cell viability. (E) After treatment with complex **1** for 48 h, A375 cell viability was detected.
